# Supplementary material for: COZOID: contact zone identifier for visual analysis of protein-protein interactions
Source: BMC Bioinformatics. 2018 Apr 6;19:125. doi: 10.1186/s12859-018-2113-6 (PMC5889581; doi:10.1186/s12859-018-2113-6)
Supplement: Supplementary file 2 — Software build. Executable binary file of the software tool. (ZIP 118,784 kb) [file 12859_2018_2113_MOESM2_ESM.zip › cozoid/java/docs/beansbinding-1.2.1-doc.zip!/org/jdesktop/el/impl/lang/class-use/EvaluationContext.html]

Uses of Class org.jdesktop.el.impl.lang.EvaluationContext (Beans Binding)


---


|  |  |  |
| --- | --- | --- |
| **Overview** \| **Package** \| **Class** \| **Use** \| **Tree** \| **Deprecated** \| **Index** \| **Help** | |  |
| PREV   NEXT | **FRAMES**    **NO FRAMES**     **All Classes** |  |


---


## **Uses of Class org.jdesktop.el.impl.lang.EvaluationContext**

| Packages that use EvaluationContext | |
| --- | --- |
| **org.jdesktop.el.impl.parser** | Customized version of EL for Beans Binding; not for general use. |

Uses of EvaluationContext in org.jdesktop.el.impl.parser

| Methods in org.jdesktop.el.impl.parser with parameters of type EvaluationContext | |
| --- | --- |
| `MethodInfo` | `SimpleNode.getMethodInfo(EvaluationContext ctx, java.lang.Class[] paramTypes)` |
| `MethodInfo` | `Node.getMethodInfo(EvaluationContext ctx, java.lang.Class[] paramTypes)` |
| `MethodInfo` | `AstValue.getMethodInfo(EvaluationContext ctx, java.lang.Class[] paramTypes)` |
| `MethodInfo` | `AstIdentifier.getMethodInfo(EvaluationContext ctx, java.lang.Class[] paramTypes)` |
| `java.lang.Class` | `SimpleNode.getType(EvaluationContext ctx)` |
| `java.lang.Class` | `Node.getType(EvaluationContext ctx)` |
| `java.lang.Class` | `BooleanNode.getType(EvaluationContext ctx)` |
| `java.lang.Class` | `AstValue.getType(EvaluationContext ctx)` |
| `java.lang.Class` | `AstString.getType(EvaluationContext ctx)` |
| `java.lang.Class` | `AstNull.getType(EvaluationContext ctx)` |
| `java.lang.Class` | `AstNot.getType(EvaluationContext ctx)` |
| `java.lang.Class` | `AstNegative.getType(EvaluationContext ctx)` |
| `java.lang.Class` | `AstLiteralExpression.getType(EvaluationContext ctx)` |
| `java.lang.Class` | `AstInteger.getType(EvaluationContext ctx)` |
| `java.lang.Class` | `AstIdentifier.getType(EvaluationContext ctx)` |
| `java.lang.Class` | `AstFunction.getType(EvaluationContext ctx)` |
| `java.lang.Class` | `AstFloatingPoint.getType(EvaluationContext ctx)` |
| `java.lang.Class` | `AstEmpty.getType(EvaluationContext ctx)` |
| `java.lang.Class` | `AstDynamicExpression.getType(EvaluationContext ctx)` |
| `java.lang.Class` | `AstDeferredExpression.getType(EvaluationContext ctx)` |
| `java.lang.Class` | `AstCompositeExpression.getType(EvaluationContext ctx)` |
| `java.lang.Class` | `AstChoice.getType(EvaluationContext ctx)` |
| `java.lang.Class` | `ArithmeticNode.getType(EvaluationContext ctx)` |
| `java.lang.Object` | `SimpleNode.getValue(EvaluationContext ctx)` |
| `java.lang.Object` | `Node.getValue(EvaluationContext ctx)` |
| `java.lang.Object` | `AstValue.getValue(EvaluationContext ctx)` |
| `java.lang.Object` | `AstTrue.getValue(EvaluationContext ctx)` |
| `java.lang.Object` | `AstString.getValue(EvaluationContext ctx)` |
| `java.lang.Object` | `AstPlus.getValue(EvaluationContext ctx)` |
| `java.lang.Object` | `AstOr.getValue(EvaluationContext ctx)` |
| `java.lang.Object` | `AstNull.getValue(EvaluationContext ctx)` |
| `java.lang.Object` | `AstNotEqual.getValue(EvaluationContext ctx)` |
| `java.lang.Object` | `AstNot.getValue(EvaluationContext ctx)` |
| `java.lang.Object` | `AstNegative.getValue(EvaluationContext ctx)` |
| `java.lang.Object` | `AstMult.getValue(EvaluationContext ctx)` |
| `java.lang.Object` | `AstMod.getValue(EvaluationContext ctx)` |
| `java.lang.Object` | `AstMinus.getValue(EvaluationContext ctx)` |
| `java.lang.Object` | `AstLiteralExpression.getValue(EvaluationContext ctx)` |
| `java.lang.Object` | `AstLessThanEqual.getValue(EvaluationContext ctx)` |
| `java.lang.Object` | `AstLessThan.getValue(EvaluationContext ctx)` |
| `java.lang.Object` | `AstInteger.getValue(EvaluationContext ctx)` |
| `java.lang.Object` | `AstIdentifier.getValue(EvaluationContext ctx)` |
| `java.lang.Object` | `AstGreaterThanEqual.getValue(EvaluationContext ctx)` |
| `java.lang.Object` | `AstGreaterThan.getValue(EvaluationContext ctx)` |
| `java.lang.Object` | `AstFunction.getValue(EvaluationContext ctx)` |
| `java.lang.Object` | `AstFloatingPoint.getValue(EvaluationContext ctx)` |
| `java.lang.Object` | `AstFalse.getValue(EvaluationContext ctx)` |
| `java.lang.Object` | `AstEqual.getValue(EvaluationContext ctx)` |
| `java.lang.Object` | `AstEmpty.getValue(EvaluationContext ctx)` |
| `java.lang.Object` | `AstDynamicExpression.getValue(EvaluationContext ctx)` |
| `java.lang.Object` | `AstDotSuffix.getValue(EvaluationContext ctx)` |
| `java.lang.Object` | `AstDiv.getValue(EvaluationContext ctx)` |
| `java.lang.Object` | `AstDeferredExpression.getValue(EvaluationContext ctx)` |
| `java.lang.Object` | `AstCompositeExpression.getValue(EvaluationContext ctx)` |
| `java.lang.Object` | `AstChoice.getValue(EvaluationContext ctx)` |
| `java.lang.Object` | `AstBracketSuffix.getValue(EvaluationContext ctx)` |
| `java.lang.Object` | `AstAnd.getValue(EvaluationContext ctx)` |
| `java.lang.Object` | `SimpleNode.invoke(EvaluationContext ctx, java.lang.Class[] paramTypes, java.lang.Object[] paramValues)` |
| `java.lang.Object` | `Node.invoke(EvaluationContext ctx, java.lang.Class[] paramTypes, java.lang.Object[] paramValues)` |
| `java.lang.Object` | `AstValue.invoke(EvaluationContext ctx, java.lang.Class[] paramTypes, java.lang.Object[] paramValues)` |
| `java.lang.Object` | `AstIdentifier.invoke(EvaluationContext ctx, java.lang.Class[] paramTypes, java.lang.Object[] paramValues)` |
| `boolean` | `SimpleNode.isReadOnly(EvaluationContext ctx)` |
| `boolean` | `Node.isReadOnly(EvaluationContext ctx)` |
| `boolean` | `AstValue.isReadOnly(EvaluationContext ctx)` |
| `boolean` | `AstIdentifier.isReadOnly(EvaluationContext ctx)` |
| `boolean` | `AstDynamicExpression.isReadOnly(EvaluationContext ctx)` |
| `boolean` | `AstDeferredExpression.isReadOnly(EvaluationContext ctx)` |
| `void` | `SimpleNode.setValue(EvaluationContext ctx, java.lang.Object value)` |
| `void` | `Node.setValue(EvaluationContext ctx, java.lang.Object value)` |
| `void` | `AstValue.setValue(EvaluationContext ctx, java.lang.Object value)` |
| `void` | `AstIdentifier.setValue(EvaluationContext ctx, java.lang.Object value)` |
| `void` | `AstDynamicExpression.setValue(EvaluationContext ctx, java.lang.Object value)` |
| `void` | `AstDeferredExpression.setValue(EvaluationContext ctx, java.lang.Object value)` |

---


|  |  |  |
| --- | --- | --- |
| **Overview** \| **Package** \| **Class** \| **Use** \| **Tree** \| **Deprecated** \| **Index** \| **Help** | |  |
| PREV   NEXT | **FRAMES**    **NO FRAMES**     **All Classes** |  |


---
